# Supplementary material for: Evaluation of the medically necessary, time sensitive triage score during and beyond the local COVID-19 pandemic in the Gynaecologic Oncology Unit of a tertiary hospital in South Africa
Source: PLoS One. 2023 Apr 13;18(4):e0284177. doi: 10.1371/journal.pone.0284177 (PMC10101405; doi:10.1371/journal.pone.0284177)
Supplement: Attachment — Submitted filename: renamed_8cb46.docx [file pone.0284177.s002.docx]

Congratulations to the authors for their interesting study reporting usefulness of the MeNTS score in  prioritizing patients for surgery in gynaecologic oncologic specialty .

As the authors of the study clearly demonstrate, the MENTS score is a good tool to prioritize resources in times when they are restricted in pandemic situations. The results of this paper confirm for the first time that it is applicable to more specialized units within the gynecology specialty, such as oncology.

In general, I agree with the introduction, with the description of material and statistical method, presentation of results and conclusions.

However, I want to make the following comment that I think should be put as a limitation to the study in the final part of the discussion. The authors find a clear difference in the total MENTS score, and therefore conclude that it is a valid tool to discriminate the patients who will undergo surgery; however, if we analyze each factor of the MENTS score separately, we can observe that there are very significant differences in disease and patient factor, and none in the procedure. These very marked differences in these two factors can weigh heavily on the overall MENTS score. The explanation that could be given is that the oncological pathology and morbidity were significantly higher in the operated group This situacion is commonly seen in specialized oncological units.

Except for this comment, which should be cited as a limitation of the study, it is shown that the MENTS score is a useful tool in the selection and prioritization of patients who must be operated in times of resource restriction. For all of the above, I believe that this paper meets the characteristics to be published in PLOS-ONE.
