# Supplementary material for: Evaluation of the medically necessary, time sensitive triage score during and beyond the local COVID-19 pandemic in the Gynaecologic Oncology Unit of a tertiary hospital in South Africa
Source: PLoS One. 2023 Apr 13;18(4):e0284177. doi: 10.1371/journal.pone.0284177 (PMC10101405; doi:10.1371/journal.pone.0284177)
Supplement: Attachment — Submitted filename: Response To Reviewer PLoS ONE.docx [file pone.0284177.s003.docx]

8^th^ March 2023

The Editor,

PLOS ONE Journal.

Dear Editor,

**REBUTTAL LETTER**

Thank you for the thorough review of our manuscript and for the consideration of this study in your reputable journal.

We have critically examined the comments and suggestions and each of the raised issues is addressed below;

**Editor’s comments**

1. **Comment:** Please ensure that your manuscript meets PLOS ONE's style requirements, including those for file naming on PLOS ONE’s style

**Response:** The manuscript has been reviewed to reflect PLOS ONE’s style

2. **Comment**: Please provide additional details regarding participant consent

**Response:** The need for participant consent was waived by the Research Ethics Committee as it was a retrospective study. Patients medical records were retrieved but all the data retrieved were fully anonymized.

3. **Comment:** on financial disclosure

**Response:** The authors did not receive specific funding for this work. This statement has been included in the cover letter

4. **Comment:** on competing interests

**Response:** The authors have declared that no competing interest exists. This statement has been included in the cover letter and in the online submission form.

5. **Comment**: In your Data Availability statement, you have not specified where the minimal data set underlying the results described in your manuscript can be found

**Response:** The minimal data set used to arrive at the results in this study is submitted as supporting document with this manuscript. Any other enquiry can be directed to the corresponding author. This statement has also been included in the cover letter.

6. **Comment**: Please ensure that you include a title page within your main document. You should list all authors and all affiliations as per our author instructions and clearly indicate the corresponding author

**Response:** A title page with the list of the authors and their affiliations as per PLOS ONE’s author instructions has been included within the main document.

7. **Comment**: Please review your reference list to ensure that it is complete and correct. If you have cited papers that have been retracted, please include the rationale for doing so in the manuscript text, or remove these references and replace them with relevant current references

**Response:** The authors went through the reference list, the list is complete and correct, except for the name of the third author in reference 15 which was wrongly spelt as “de le Cruz” instead of “de la Cruz”. This has been corrected in the main document.

None of the reference list has been retracted to the best of the authors’ search.

**Reviewers’ comments**

8. **First reviewer’s comment:** I want to make the following comment that I think should be put as a limitation to the study in the final part of the discussion. The authors find a clear difference in the total MENTS score, and therefore conclude that it is a valid tool to discriminate the patients who will undergo surgery; however, if we analyse each factor of the MENTS score separately, we can observe that there are very significant differences in disease and patient factor, and none in the procedure. These very marked differences in these two factors can weigh heavily on the overall MENTS score. The explanation that could be given is that the oncological pathology and morbidity were significantly higher in the operated group This situation is commonly seen in specialized oncological units.

**Response:** These statements have been added to the final part of the discussion: “The retrospective design of this study posed some limitations as it was difficult to draw accurate validation of the triaging tool. The bias with this design was however reduced by scoring each participant by two different observers. Also, the significant differences observed in both the disease and patient factors among those operated and those whose surgery was postponed could have impacted significantly on the overall MeNTS score. This could be explained by the significant higher numbers of oncological pathology and morbidity among the operated group”.

9. **Second Reviewer’s comment**: There are a few typographical errors which should be corrected. The use of the word non-emergent is inappropriate and should be replaced with non-urgent. The authors evaluated the MeNTS in Gynaecologic Oncologic settings but not describe it. It was done by Prachand et al. They should delete this from the abstract and body.

**Response:** The write up has been thoroughly perused and corrections of the few typographical errors have been made. The word non-emergent has been replaced with non-urgent. The use of the word “describe” has been replaced with “evaluate” in the abstract and body of the manuscript as suggested by the reviewer

All other corrections are shown in track changes in the main manuscript.

We anticipate a favorable response from you at your earliest convenience.

Thank you.

Yours sincerely,

Sajo AE (Corresponding author)
